# Investigating In Situ Expression of c-MYC and Candidate Ubiquitin-Specific Proteases in DLBCL and Assessment for Peptidyl Disruptor Molecule against c-MYC-USP37 Complex

**DOI:** 10.3390/molecules28062441

**Published:** 2023-03-07

**Authors:** Durr e Sameen Kamran, Mushtaq Hussain, Talat Mirza

**Affiliations:** 1 Department of Pathology, Dow Ishrat-ul-Ebad Khan Institute of Oral Health Sciences, Dow University of Health Sciences, Karachi 75330, Pakistan; 2 Bioinformatics and Molecular Medicine Research Group, Dow Research Institute of Biotechnology and Biomedical Sciences, Dow College of Biotechnology, Dow University of Health Sciences, Karachi 75330, Pakistan; 3 Department of Research, Ziauddin University, Karachi 75000, Pakistan

**Keywords:** c-MYC, DLBCL, ABC, GCB, USP37, peptidyl disruptor

## Abstract

Diffuse Large B-Cell Lymphoma (DLBCL) is the most common form of non-Hodgkin’s lymphoma (NHL). Elevated expression of c-MYC in DLBCL is associated with poor prognosis of the disease. In different cancers, c-MYC has been found regulated by different ubiquitin-specific proteases (USPs), but to date, the role of USPs in c-MYC regulation has not been investigated in DLBCL. In this study, in situ co expression of c-MYC and three candidates USPs, USP28, USP36 and USP37, have been investigated in both the ABC and GCB subtypes of DLBCL. This shows that USP37 expression is positively correlated with the c-MYC expression in the ABC subtype of DLBCL. Structurally, both c-MYC and USP37 has shown large proportion of intrinsically disordered regions, minimizing their chances for full structure crystallization. Peptide array and docking simulations has shown that N-terminal region of c-MYC interacts directly with residues within and in proximity of catalytically active C19 domain of the USP37. Given the structural properties of the interaction sites in the c-MYC-USP37 complex, a peptidyl inhibitor has been designed. Molecular docking has shown that the peptide fits well in the targeted site of c-MYC, masking most of its residues involved in the binding with USP37. The findings could further be exploited to develop therapeutic interventions against the ABC subtype of DLBCL.

## 1. Introduction

Diffuse Large B-cell Lymphoma (DLBCL) is the most common form of non-Hodgkin’s lymphoma (NHL), accounting for nearly 40% of the NHL cases [1,2]. The WHO classifies DLBCL according to cells of origin and genome wide expression profiling (GEP) into two primary subtypes, namely Germinal Center B-cell-like (GCB) and Activated B-Cell-like (ABC/non-GCB). Both subgroups have different mutational profiles, oncogenic pathways, responses to treatment and prognoses [3]. As GEP studies are not commonly available in diagnostic setups and if available are not cost effective, the WHO classification emphasizes on the role of immunohistochemistry (IHC) for classifying DLBCL based on Han’s algorithm [4]. The algorithm differentiates DLBCL as GCB/ABC based on differential expression of CD10, MUM1, BCL2 and BCL6 immuno-histochemical stains [5]. The GCB subgroup accounts for almost half the cases of DLBCL in comparison to the ABC subtype, which accounts for 30% cases of DLBCL, while the remaining 20% of the cases are considered not otherwise classified DLBCL [5,6]. The GCB subtype of DLBCL has shown better prognosis and survival rates compared to the ABC subtype of the lymphoma [7].

Similar to many other cancers, genetic aberrations, mutation and translocations are also associated with DLBCL [1,8]. Chromosomal translocations in DLBCL lead to homotopic (BCL6) or heterotopic (MYC and BCL2) dysregulated expression of the proteins [9]. Among these, dysregulation of c-MYC holds profound importance. c-MYC is a master transcriptional factor, estimated to be involved in the regulation of the expression of nearly 15% of the human genome [10]. Many target genes of c-MYC are critically important in cellular transformation, cell cycle and apoptosis, and often dubbed as proto-oncogenes [11,12,13]. Expressional dysregulation of c-MYC has also been observed in several hematological malignancies, including DLBCL [14,15,16,17], and its over expression has been linked to poor prognosis of the disease [18,19,20].

Expression of c-MYC is post-translationally regulated by different ubiquitin-specific proteases (USPs) by directly interacting with c-MYC and stabilizing the protein from proteosomal degradation. In different cancers, cellular turnover of c-MYC has been regulated by different USPs [21,22,23,24,25,26,27,28], with notable examples including the maintenance of c-MYC turnover by direct physical interactions of USP28 in colon carcinoma [22], USP36 in breast carcinoma [23] and USP37 in lung cancer [27]. Nevertheless, despite expression of c-MYC having been found consistently dysregulated in DLBCL [9,12,29], the role of USPs in this regard is largely unknown. This study aims to explore to monitor the expression of c-MYC and its three major USPs, namely USP28, USP36 and USP37, in DLBCL subtypes. Additionally, physical interaction points between c-MYC and targeted USP have been predicted using bioinformatics tools. This enables us to predict and propose the disruptor peptides that could be used for the therapeutic interventions.

## 2. Results

### 2.1. Typing of DLBCL Cases

**Figure 1:**
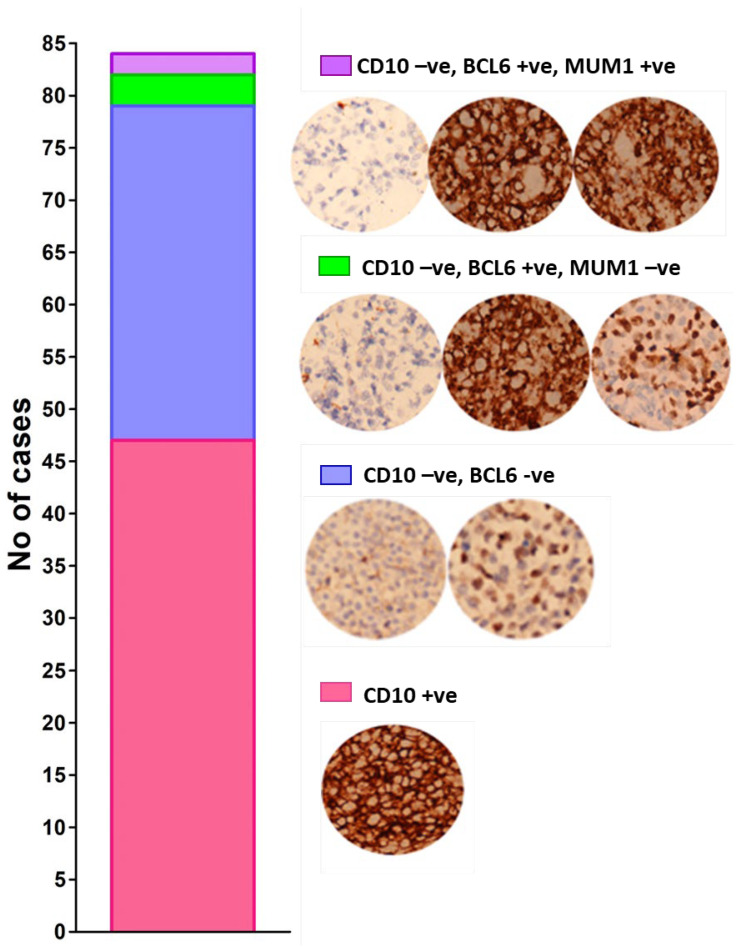
Stacked bar showing the distribution of expression of markers in the recruited DLBCL cases. Representative micrographs of CD10 + ve GCB (**Top**), CD10-ve, BCL-6-ve ABC (2nd) CD10-ve, BCL-6 + ve, MUM1-ve (3rd) and CD10-ve, BCL-6 + ve and MUM1 + ve (**bottom**) are also shown, corresponding to the colors of the stacks.

Subtyping of DLBCL as GCB and ABC was carried out on the basis of Han’s algorithm [4]. Cases which showed expression of CD10 were identified as GCB, whereas those that appeared negative for CD10 were further classified on the basis of expression of BCL6 and MUM1. Cases with positive expression of BCL6 and negative for MUM1 are also designated as the GCB subtype of DLBCL. Conversely, all other permutations of the expression are regarded as the ABC subtype of DLBCL. Out of 84 samples assessed, 50 were classified as GCB subtype, whereas 34 cases were classified as ABC subtype of DLBCL (Figure 1), suggesting slightly more common incidence of the GCB subtype of DLBCL compared to the ABC subtype among the screened tissues.

### 2.2. Expression Analysis

**Figure 2:**
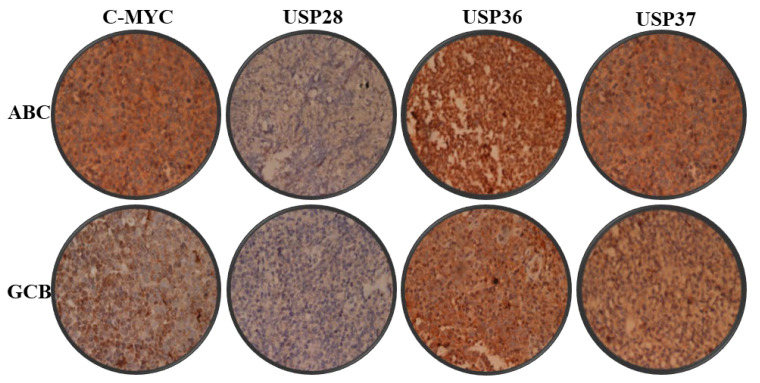
Immunohistochemistry of DLBCL samples. Representative histomicrographs (400×) of the ABC and GCB subtypes of DLBCL probed with c-MYC, USP28, USP36 and USP37 antibodies as labeled.

**Figure 3:**
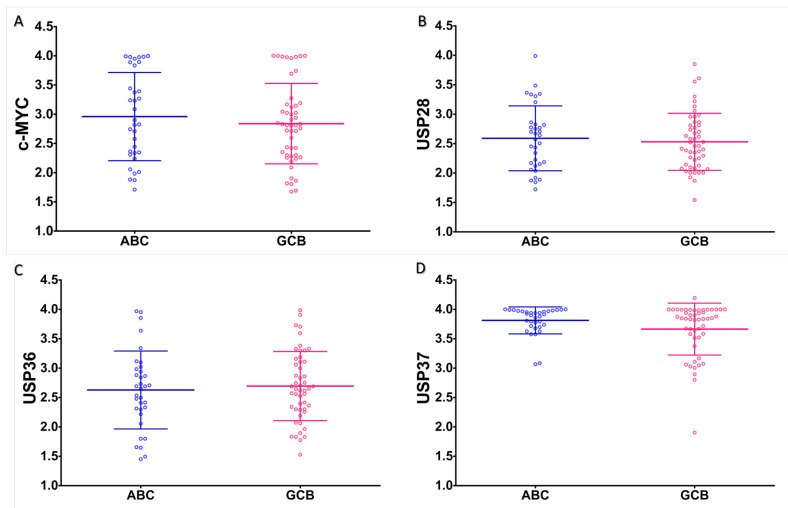
Expression of c-MYC and USPs in subtypes of DLBCL. Graphs showing expression (OD) of (**A**) c-MYC, (**B**) USP28, (**C**) USP36 and (**D**) USP37 in ABC (Blue) and GCB (Red) subtypes of DLBCL. Small and large horizontal lines represent mean and standard deviation, respectively.

Expression of c-MYC and associated USPs were estimated digitally from immunohistomicrographs (Figure 2). Expression of all examined USPs and c-MYC was found comparable in both the ABC and GCB subtypes of DLBCL without any statistically significant difference (Figure 3).

### 2.3. Correlation Analysis

**Figure 4:**
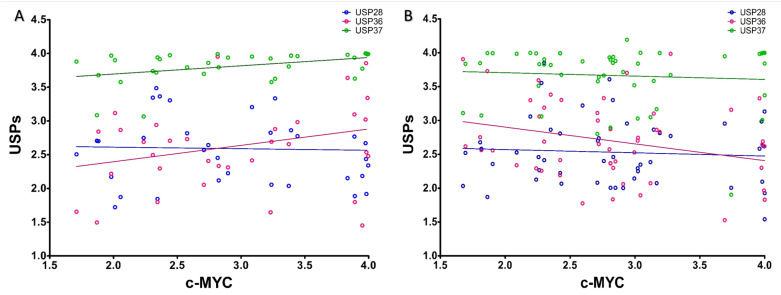
Correlation between c-MYC and USPs expression. Correlation between expression of c-MYC and USPs, USP28 (Blue), USP36 (Red) and USP37 (Green) are shown in the (**A**) ABC and (**B**) GCB subtypes of DLBCL. Data points are indicated with the empty circles of corresponding colors.

Correlation analysis between the expression of c-MYC and its stabilizers, USP28, USP36 and USP37 showed statistically significant positive association of USP 37 with c-MYC in case of the ABC subtype (*p* = 0.0184; r^2^ = 0.1616). In comparison, the association between c-MYC and USP28 (*p* = 0.8580; r^2^ = 0.001016) and c-MYC and USP36 (*p* = 0.1139; r^2^ = 0.07626) expressions were found statistically insignificant in the ABC subtype of DLBCL (Figure 4A). However, in the GCB subtype of DLBCL, negative association between the expression of c-MYC and USP36 was observed (*p* = 0.0414; r^2^ = 0.08387). Conversely, the association between c-MYC and USP28 (*p* = 0.6402; r^2^ = 0.004588) and c-MYC and USP37 (*p* = 0.08387; r^2^ = 0.006006) expressions were found to be statistically insignificant in the GCB subtype of DLBCL (Figure 4B). This implies a subtype-based selective and/or differential involvement of USPs in the regulation of c-MYC turnover in DLBCL, where USP37 in particular is involved in regulating the turnover of c-MYC in the ABC subtype of DLBCL.

### 2.4. Physical Mapping

**Figure 5:**
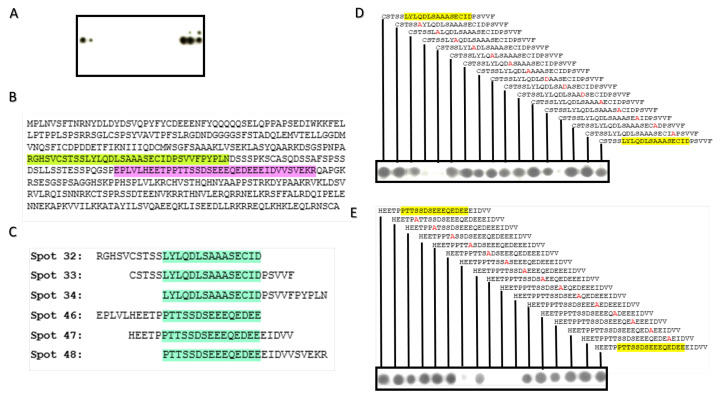
Physical mapping of c-MYC for USP 37 interaction. (**A**) Peptide array of c-MYC protein probed with USP37 showing binding of the protein with the 25 mers spots (32–34 and 46–48) of c-MYC. (**B**) Protein sequence of c-MYC showing regions corresponding to spots 32–34 (highlighted lime green) and spots 46–48 (highlighted fuchsia pink). (**C**) Peptide sequence spotted on each binding spots with consensus regions highlighted aqua). Alanine scan array of consensus sequence (highlighted yellow) in (**D**) spot 33 and (**E**) spot 47 of c-MYC. Black dots showing effective binding of the c-MYC peptide with USP37, whereas lighter or absence of the dots represent weak or absent binding due to the substitution of the residue with alanine/aspartic acid (red colored) in c-MYC alanine array.

Since stabilization of c-MYC by different USPs in different cancers is underpinned by direct physical interaction between both molecules [22,23,27] including USP37 [27], it is, therefore, also conceivable that in the ABC subtype of DLBCL, c-MYC is likely regulated by direct interaction with USP37. Considering this, we explored the physical interaction site of c-MYC for USP37 interaction by using peptide array. Probing of c-MYC peptide array with recombinant USP37 showed six spots of positive binding corresponding to two different physical regions, Arg166-Asn200 and Glu236-Arg270, of c-MYC (Figure 5A,B). From this, the consensus regions of the c-MYC for USP37 interaction was derived as Leu176-Asp190 and Pro245-Glu260 (Figure 5C).

### 2.5. Alanine Scan

To further resolve the interaction points, alanine scans of consensus regions were probed with recombinant USP37. Alanine scans of both the consensus regions showed inhibition in the interaction between c-MYC and USP37 when the residues Tyr177, Gln179, Asp180, Cys188, Asp251, Glu253, Glu254 and Glu255 were substituted with alanine (Figure 5D,E).

### 2.6. Predicting c-MYC and USP37 Interaction

**Figure 6:**
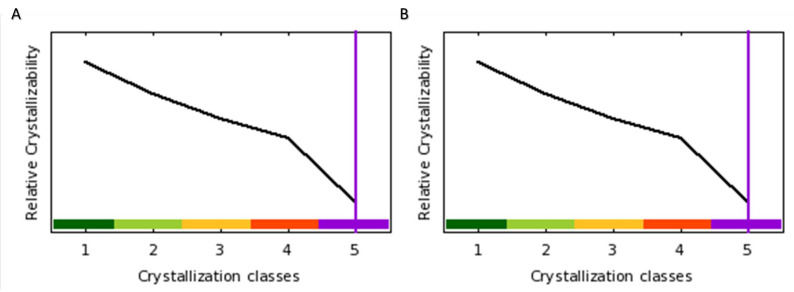
Protein crystalizability potential of (**A**) c-MYC and (**B**) USP37. Here, the x axis represents the increasing difficulty in crystallization and the vertical purple line represent the score of the molecule.

Since stabilization of c-MYC by USP37 is unwanted, disrupting the physical interaction between c-MYC and USP37 has therapeutic potential. However, neither of these molecules are structurally resolved to their full extent. Assessment of c-MYC and USP37 showed least probability of crystallization (Figure 6). Low crystalizability of c-MYC is due to its length (439 a.a), span of structurally disordered regions (165 residues), instability index (92.28), high percentage of coiled structures (71), coiled coils (53), insertion score (0.32) and surface hydrophobicity (−0.81). In comparison, the low potential of USP37 to be crystalized is mainly due to its large size (979 a.a.), extensive proportion of disordered region (297 residues), instability index (58.94), percentage of coiled structures (65), coiled coils (53), insertion score (0.16), surface hydrophobicity (−0.92) and surface ruggedness (1.13). Collectively, these values indicate that it is difficult to resolve the structures of these molecules empirically. 

### 2.7. Molecular Model of c-MYC

**Figure 7:**
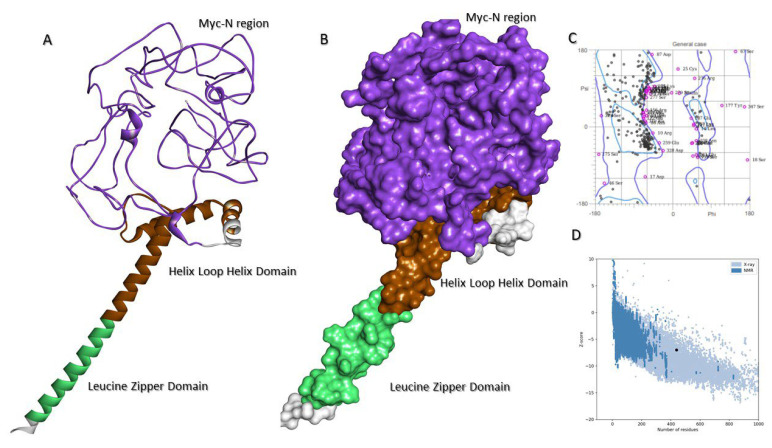
Molecular structure of c-MYC. (**A**) Full-length ribbon structure of c-MYC, where domains are colored differently: MYC-N region (purple), Helix Loop Helix (brown) and Leucine Zipper (aqua). (**B**) Surface topology of c-MYC structure with domains are colored correspondingly. (**C**) Ramachandran plot and (**D**) ProSA graph are shown, where black spots in the ProSA graph indicate z scores of the modeled structure over the structures, resolved experimentally by NMR (dark blue) and X-ray crystallography (light blue).

Since it is difficult to crystallize both c-MYC and USP37, we adopted molecular modeling to investigate 3D structural conformation of both proteins. PDB blast showed that only the C-terminal (Asn353-Leu434) of c-MYC (PDB id: 1NKP), comprising helix loop helix and leucine zipper subdomains, is resolved. Therefore, the full-length molecular model of c-MYC was constructed (Figure 7A,B), which retained the C-terminal structural conformation compared to the resolved structure (RMSD 1.10 Å), whereas the N-terminal portion of protein was found intrinsically disordered. The ProSA Z score (−6.99) and distribution of dihedral angles in the Ramachandran Plot showed structural plausibility of the constructed model (Figure 7C,D).

### 2.8. Molecular Model of USP37

**Figure 8:**
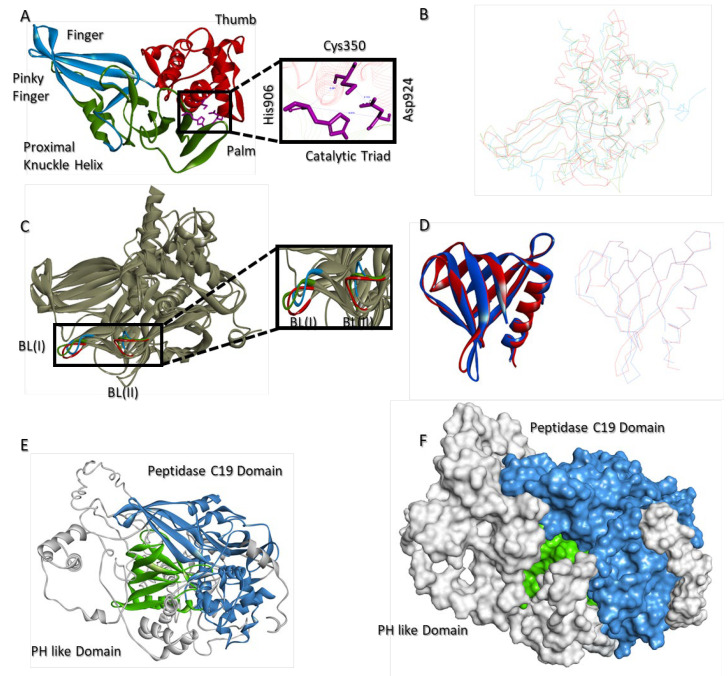
Structure of USP37. (**A**) Ribbon diagram of the C19 domain of USP37 is shown, where structural subdomains are labeled and represented with different colors along with a catalytic triad. (**B**) Superimposition of the Cα backbone of the C19 domain of USP37 (red) over the C19 domain of USP12 (blue) and USP46 (green). (**C**) Superimposition of blocking loops I and II of USP37 (red) over the BL of USP12 (blue) and USP46 (green) in the full C19 ribbon conformation. (**D**) Superimposition of the structurally resolved PH domain (blue) over the modeled PH domain of USP37 in a full-length model (red) in ribbon and Cα wire conformations. (**E**) Full-length ribbon structure and (**F**) surface topology of USP37, where domains are labeled and colored differently.

PDB-BLAST of USP37 revealed that only a partial structure (Leu4 to Gly125; PDBid: 3U12), containing the Pleckstrin Homology (PH) domain of USP37, is structurally resolved. Importantly, the major functional and catalytically active C19 domain (Phe342-Thr603 and Glu881-Met949) of the protein was unresolved. Among the known structures of C19 domain, USP37 C19 domain shows maximum sequential identity (29%) with both USP12 (PDBid:5K16) and USP46 (PDBid:5L8H). The molecular conformation of C19 domain resembles with canonical right hand like folds of USPs and is divided into three characteristic structural subdomains, namely fingers (479–519 and 531–555 a.a.), thumb (342–478 a.a.) and palm (520–530 and 556–949 a.a). Similar to USP12 and USP46, the finger domain of USP37 C19 also has four antiparallel β sheets, with the pinky finger ranging from Glu532 to Ser537. However, unlike USP12 and USP46 structures, where it appears as a coiled structure, in USP37, the pinky finger adopts proper β sheet conformation. Consistent with USP12 and USP46 structures, a bridging α helix (Ser520-Arg530) is found between the fingers and palm structural domains of C19 of USP37, referred to as the Proximal Knuckle (PK) helix. Catalytic triad (Cys350, His906 and Asp924) is located at the interface of the thumb and palm. The distance between Cys350 and His906 and Cys350 and Asp924 is 6.661 Å and 6.153 Å, respectively, whereas the distance between His906 and Asp924 is 9.979 Å (Figure 8A). Superimposition of USP37 with most homologous C19 structures of USP12 and USP46 shows RMSD values of 1.33 Å and 1.43 Å, respectively (Figure 8B). Similarly, blocking loop I (Leu574-Asn569) and II (Ser900-Gly905) are also present in the C19 domain of USP37; however, their spatial positioning especially of blocking loop I is profoundly different in USP37 compared to USP12 and USP46 (Figure 8C). In addition to C19 domain, the N-terminal of USP37 also has an additional PH domain (Leu4-Arg106). The domain was previously resolved by X-ray crystallography (PDBid:3U12). The domain modeled in the full-length proposed structure of USP37 shares close structural homology with the independently resolved structure, as reflected by a Cα deviation of 0.93 Å (Figure 8D). Similar to the resolved structure, the PH domain in the full-length model of USP37 has four antiparallel β sheets blocked by C terminal α helix. In the three-dimensional conformation, PH domain of USP37 is located right above the interface of the fingers and thumb (Figure 8E,F).

### 2.9. c-MYC-USP37 Intermolecular Complexes

**Figure 9:**
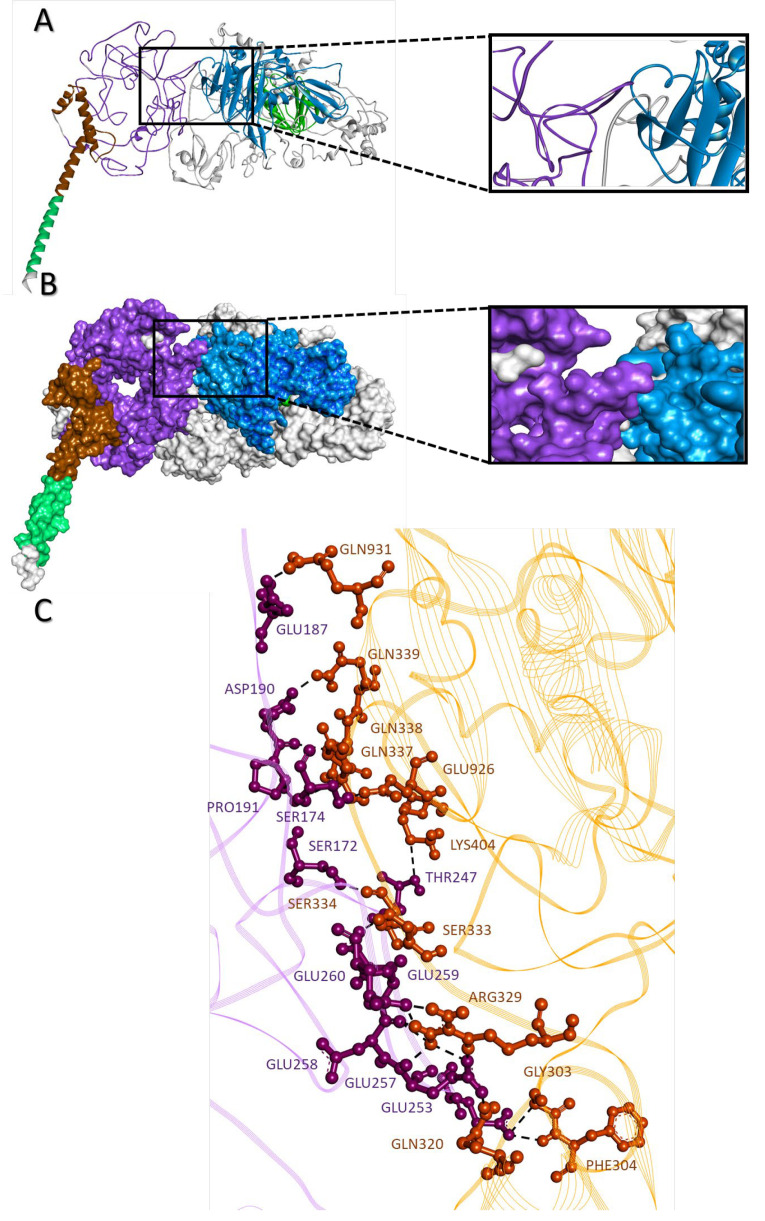
c-MYC-USP37 molecular complex. Intermolecular complexes between c-MYC and USP37 are shown in (**A**) ribbon configuration and (**B**) surface topology along with the magnified interaction regions. (**C**) Ball and stick representation of intermolecular interactions between amino acids of c-MYC (purple) and USP37 (orange), where the dotted lines represent intermolecular hydrogen bonds.

Molecular docking between USP37 and c-MYC models predicted direct physical interaction between N-terminal of the c-MYC with the residues at and around the C19 domain of USP37. Dissociation constant and free energy of the c-MYC-USP37 molecular complex was predicted to be 1.9 × 10^−8^ Kda and −8.1 KJ, respectively. Broadly, intermolecular interactions were dominated by charged residues and classified as charged-charged (8), charged-polar (16), charged-apolar (21), polar-polar (11), polar-aploar (10) and apolar-apolar (2) types (Figure 9A–C).

### 2.10. Disrupting c-MYC-USP37 Complex

**Figure 10:**
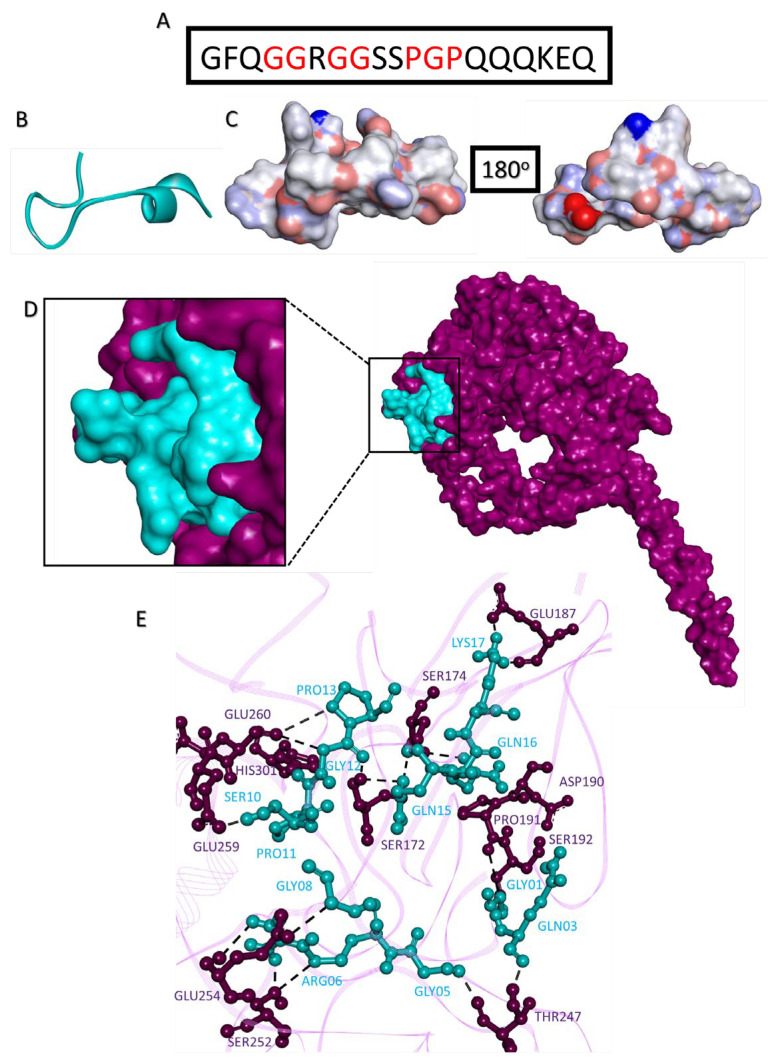
Disruptor peptide for the c-MYC-USP37 complex. (**A**) Primary sequence of designed disruptor peptide. Structure of disruptor peptide in (**B**) ribbon configuration and (**C**) electrostatic surface topology with 180° rotated view. (**D**) Surface topology of molecular complex between c-MYC (purple) and disruptor peptide (aqua); the interaction regions have been shown in the magnified inset. (**E**) Ball and stick representation of intermolecular interactions between amino acids of c-MYC (purple) and disruptor peptide (aqua), where dotted lines represent intermolecular hydrogen bonds.

Since the molecular interactions between c-MYC and USP37 are majorly driven by the intrinsically disordered regions in both proteins, instead of a small molecule, a peptidyl disruptor was designed. The sequence was derived from the USP37 interaction points, to mask its binding regions on c-MYC, intervened with proline and glycine residues to minimize stearic hindrances and/or collision (Figure 10A–C). Structurally, the peptide was predicted to loop, and by and large, the electrostatic surface of the molecule is partially polar. HADDOCK prediction showed that peptide in majority of the simulations interacted with the c-MYC to the regions targeted (USP37 binding sites) and sits well within the cavity of c-MYC that binds with USP37. Among all, the best docking simulation selected on the basis of free energy (−11.1 kJ), dissociation constant (1.5 × 10^−8^) and intermolecular interactions showed interaction with Ser172, Ser174, Glu187, Asp190, Pro191, Ser192, Thr247, Ser252, Glu254, Glu259, Glu260 and His301 residues of c-MYC, out of which Ser172, Glu187, Asp190, Pro191, Thr247, Glu259 and Glu260 were also predicted to interact with USP37 (Figure 10D,E).

## 3. Discussion

DLBCL is one of the most aggressive forms of blood cancer and constitute nearly 40–50% of all NHL [1,2]. Out of the two major types of DLBCL, the ABC subtype is considered pathologically more detrimental and have poor prognosis compared to GBC type of DLBCL [7]. Similar to our observation, most studies estimated that GCB is relatively more common form of the DLBCL compared to the ABC [30,31,32]; however, the difference between the proportion of both major types of DLBCL is marginally small. For example, screening of 62 DLBCL cases from Pakistan estimated 56% cases of ABC compared to the GCB subtype of DLBCL [33]. However, a relatively large-scale study on 466 DLBCL patients as part of International DLBCL Rituximab-CHOP Consortium Program study [30] showed slightly higher prevalence of GCB (52%) compared to ABC (48%) subtypes of DLBCL. Consistently [31] reported higher prevalence of GCB (52.3%) compared to ABC (48.7%) subtypes of DLBCL when analyzed on total of 161 samples. Since the difference in the prevalence of the ABC and GCB subtypes is marginally small, it could be conceivable that under conventional and/or more widely employed mode of screening, both subtypes are nearly equal in their prevalence. However, relatively more refined tools such as GEP may render different results. For instance, a GEP-based investigation on 240 DLBCL cases has shown 47.9% prevalence of GCB compared to 30.4% of ABC subtypes of DLBCL [32].

Where there is a little difference in the clinical manifestations of the both the ABC and GCB subtypes of DLBCL, their clinical outcome is noticeably different [3]. This could very well be due to the difference in the molecular basis of pathology between both cancers [34]. However, c-MYC has been found a major molecular denominator that underscores the molecular pathology of the disease. Different alterations in c-MYC have been reported in DLBCL, mainly due to chromosomal translocation, expressional dysregulation, mutations and copy number variation, leading to the over expression of MYC protein [35]. Consequently, the ensuing over expression of c-MYC has been associated with the poor prognosis of the disease [16,17,29]. Although the expression of c-MYC has been observed elevated in DLBCL, no study has been found in relation to the comparison of the expression of the molecule in both the major subtypes of DLBCL. Therefore, we first sought to compare the expression of c-MYC in both subtypes of DLBCL. For this purpose, we have quantified the expression observed through IHC by ImageJ IHC profiler plugin. Previously, the digital estimation of IHC images has shown consistent and more accurate estimation of the expression of marker and/or proteins in the tissue sample compared to the eye ball assessments [36,37]. For example, digital estimations of the Her2µ, Ki67 and Pan Cytokeratin [AE1/AE3] have shown relatively better assessment of the marker expressions in breast cancer [38], neuroendocrine tumor [39] and colorectal cancers [40], respectively. Our finding showed no statistically significant difference between the expression of c-MYC between the GCB and ABC subtypes of DLBCL. Previously, difference in the expression of c-MYC and other related proteins have been observed in different stages and subtypes of other cancers. For example, concomitant decrease in the expression of c-MYC and ß-catenin has been reported by Lee et al. (2016) [41] with the advancing stages of colorectal cancers. Similarly, a study observed over expression of c-MYC in established oral squamous cell carcinoma when compared to oral epithelial dysplasia [42]. In the GEP analysis of breast cancer, five major subtypes have been established, where over expression of c-MYC has been observed in basal-like tumors with poor prognosis compared to the other subtypes, such as luminal A, luminal B, Her-2µ positive and normal-like breast cancer [43].

Over expression of c-MYC could be post translationally regulated by ubiquitin-specific proteases, by deubiquitinating the c-MYC and in turn preventing them from proteosomal degradation [21,22,23,24,25,26,27,28]. For instance, in breast carcinoma USP22 and USP36 are known to regulate the cellular turnover of c-MYC [21,23]. In colon carcinoma and lung cancer, c-MYC expression is maintained by USP28 [24] and USP37 [27], respectively. To date, no study has been reported the involvement of USPs in cellular turnover of c-MYC in DLBCL. Therefore, we statistically assess co-expression of c-MYC with three of the most widely assessed USPs and found USP37 expression is positively associated with the over expression of c-MYC in the ABC subtype of DLBCL but not with its GCB subtype. We use the *p* value as an indicator of statistical significance rather than the r^2^ value, as previously employed in several earlier studies for associating the expression of any two molecules in different cancers [44,45,46]. The positive association between c-MYC and USP37 expression in only the ABC subtype of DLBCL points to the difference in the molecular events that underscore the pathology of both types of DLBCL. For example, it has been observed that MYC translocation, BCL2 translocation and PTEN deletion are preferentially observed in the GCB subtype of DLBCL compared to the ABC subtype of DLBCL. Similarly, the ABC subtype of DLBCL is characterized by chronic active B cell receptor (BCR) signaling and NF-kB deregulation [34], which have so far not been reported in the GCB subtype of DLBCL. Additionally, it is also possible that some other paralogue of USP, such as USP13, USP16, USP17 and USP22, may be involved in the post translational maintenance of c-MYC in the GCB subtype of DLBCL [21,25,26,28]. In summary, this represents the differential mode of regulation of cellular turnover of c-MYC between the GCB and ABC subtypes of DLBCL.

USP37 has been found to regulate the turnover of c-MYC in lung carcinoma by direct physical interaction [27]. Moreover, other USPs that regulate c-MYC turnover in different cancers also tend to directly interact with c-MYC [21,22,23,24,25,26,27,28]. Therefore, it is plausible to conceive that in the ABC subtype of DLBCL the c-MYC may also interact with USP37, resulting in its stabilization. Considering this, we sought to identify the interaction points between c-MYC and USP37. For this purpose, our peptide array and alanine scan identified the interacting residues of c-MYC protein for USP37. Previously, peptide array systems have been successfully used to find the bindings sites of many molecular complexes [47,48]. Both proteins have shown low potential of crystallization, suggesting difficulty in the empirical resolution of structure of the proteins. Computational modeling has long been successfully used to predict the structure of the proteins, especially when the proteins contain significant proportion of disordered regions [49]. It has been demonstrated that proteins with high proportion of disordered region exhibit greater partner protein promiscuity. Structurally c-MYC has shown large proportion of disordered regions and this master transcriptional regulator has been known to physically interact with variety of different proteins such as TRRAP, STAGA and TIP60 [50], in addition to the DNA. DNA interaction of c-MYC is majorly based on leucine zipper and basic loop helix domains [51]. Structurally disordered N-terminal domain of the c-MYC as predicted in the study is in line with many of the functional studies showing interaction of c-MYC molecule with number of different proteins via its N-terminal region [50,52]. As expected, usually, intrinsically disordered regions of a protein physically interact with the region of same structural attributes of the partner protein [53]. This is again in line with our docking studies where intrinsically disordered regions of both USP37 and c-MYC were predicted to interact with each other. Moreover, interaction of c-MYC with USP37 is expected to remove ubiquitin tag from the protein via its catalytically active C19 domain. In the docking simulation the intrinsically disordered region of USP37 is present in the proximity of its C19 domain and some residues of the C19 domain were also predicted to interact with c-MYC. This indicates that the predicted c-MYC USP37 complex is structurally plausible.

The interaction between c-MYC and USP37 leads to post translational stabilization of the c-MYC, resulting in the abnormal increase in the half-life of the molecule, which is estimated to be 30 min in normal cells [54]. This retention of c-MYC with in cell leads to abnormal growth of the cells, and therefore, the interaction sites of c-MYC-USP37 complex could be targeted to prevent increased accumulation of c-MYC within the cells. Since the interacting region of c-MYC for USP37 is structurally disordered, it may not be a suitable target for small molecule inhibitor and/or disruptor [55]; rather, a peptidyl disruptor could be used to block the interaction between USP37 and c-MYC. Peptidyl disruptors has shown effective inhibition for the protein complex formation such as human ACE2 and the receptor-binding domain (RBD) of the Spike protein of SARS-CoV2 [56] YAP/TAZ-TEAD Transcriptional Complex [57] and Human Cytomegalovirus Core Nuclear Egress Complex [58]. Nearly 80% of the residues of c-MYC that interacts with USP37 have been found interacting with the designed peptide, suggesting that the peptide is specific to c-MYC-USP37 complex and could be used for targeted therapy against the ABC subtype of DLBCL.

## 4. Materials and Methods

### 4.1. Recruitment of Samples

The study was retrospective in nature where samples (paraffin blocks) were retrieved from surgical pathology unit of the tertiary care hospital. The samples were fixed in 10% buffered formalin, embedded in paraffin and processed in automated tissue processor EXCLESIOR AS (Thermo Scientific/Waltham, MA, USA). In total, 100 samples were included, of which 16 samples were discarded due to the lack of availability of diagnostic data or because of insufficient tissue in blocks. Finally, 84 diagnosed samples of DLBCL were included in the study. Samples were given Medical Record number (MR) in the lab to maintain confidentiality. The study protocol was approved from Institutional Review Board of the Institute.

### 4.2. Subtyping of DLBCL Cases

The subtyping of DLBCL cases into ABC and GCB subtypes was performed by routine morphology and immunohistochemistry employing Han’s Algorithm [4]. For this purpose, serial 4 µm-thick sections were cut from paraffin-embedded tissue blocks and stained with hematoxylin and eosin (HE) and examined under light microscope for routine diagnostic histopathology. All samples were also evaluated by immunohistochemical staining for CD10, MUM1, BCL2 and BCL6 (DAKO, Denmark). Each slide was then assessed by at least two histopathologists and then discussed in department consultation committee to confirm the diagnosis and subtyping of DLBCL. Traditionally, >30% of the immunohistochemical expression of the markers was considered positive as recommended by the Han’s algorithm.

### 4.3. In Situ Expression of c-MYC and Candidate USPs

The retrieved blocks were subjected to microtomy (4 µm) and at least seven subsequent sections were fixed on lysine coated slides. The tissue sections were first treated twice with xylene for 10 min for de-paraffinization, then immersed into serially decreasing concentrations (100%, 95% and 70%) of alcohol for 3 min each. The sections were then washed by deionized water. Antigen retrieval was achieved by microwaving the slides in sodium citrate buffer followed by incubation of slides for 10 min in 3% hydrogen peroxide. This was followed by the treatment with blocking solution (animal serum/albumin) for 10 min. The sections were then rinsed multiple times with tap water followed by washing with Tris Buffer Saline with Tween-20 (TBST). Later, the slides were incubated with primary antibodies (ThermoFisher, Waltham, MA, USA) of c-MYC (MA1-980;1:90), USP28 (PA5-50858; 1:100), USP36 (PA5-50154; 1:80) and USP37 (PA5-60939: 1:80) for 20 min at room temperature, washed with PBS (Phosphate Buffer Saline) and incubated with their respective secondary antibodies for 20 min. This was then followed by incubation in DAB (Diaminobenzidine) chromogen for 10 min at room temperature and counter stained with hematoxylin and dehydration of tissue sections with increasing concentration of alcohol. Clearing of sections was finally achieved by xylene: phenol mixture (1:1), 2 changes of xylene for two minutes each and finally mounted with DPX (BDH).

### 4.4. Digital Imaging and Estimation of Immunohistochemical Stains Expression

All slides were examined under light microscope Nikon (Tokyo, Japan) Eclipse80i and 400× magnification is used for capturing images. For c-MYC and USPs, three representative fields were taken. The images were properly labeled with the MR no. and were saved as jpeg image. The images were then imported to the Image J software augmented with IHC profiler plug-in [39]. Expression of c-MYC and USPs was assessed by plotting a histogram profile of the deconvoluted DAB image under low positive, positive and high positive log scoring criteria. Finally, Optical Density score (OD) was deduced using the following formula [59]:


$$\frac{\left(\text{\% high positive }\times \;4\;+\text{ \% positive }\times \;3\;+\text{ \% low positive }\times \;2\;+\text{ \% negative }\times \;1\right)}{100}$$

### 4.5. Cloning of USP37

Briefly, cDNA of USP37 (Ala101-His430) was cloned in pet28c expression vector at HindIII and XhoI site and transformed into *E. coli BL2* strain. The protein was expressed by growing the transformed *E. coli* in LB broth with IPTG induction. Bacterial growth was separated by centrifugation after 24 h of incubation and lysed using Bug Buster (Novagen, 71456-4). The recombinant protein was then purified using Ni slurry (Qiagen, 1018244) and expression of the protein was verified by western blotting.

### 4.6. Peptide and Alanine Array Scanning

The membrane peptide array and alanine scan array were commercially synthesized. Peptide array of c-MYC covered the entire length of the protein in the form of 25 mer peptide spot with five amino acid shift from N to C terminal. In the alanine scan array, selected peptides were spotted where each amino acid was substituted by alanine or aspartic acid (in case of alanine present in native peptide). Array membranes were prepared by first blocking using 5% not fat milk (NFM) solution for four hours. After blocking, the arrays were probed with recombinant USP37 protein in probing buffer (1% NFM in PBST) for overnight at 4 °C. Finally, the arrays were washed thrice with 1xTBST for 10 min and probed with the 6x His tag antibodies (abcam, ab1187) for two hours at room temperature. Arrays were washed thrice with 1xTBST at room temperature for 10 min and developed using ECL detection kit.

### 4.7. Bioinformatic Analysis

#### 4.7.1. Sequence Retrieval and Domain Identification

Full length protein sequences of c-MYC protein and USP 37 were retrieved from the UniProt database [60]. The sequences were subjected to PDB Blast to retrieve the homologous protein which have been structurally resolved and deposited at RCSB database [61]. This could further be used as template during modeling of c-MYC and USP37, correspondingly. For the identification of different domains and functionally active sites in the proteins, Conserved Domain Database (CDD) of NCBI [62] and UniProt database were used [60].

#### 4.7.2. Protein Crystallizability Potential

For predicting protein crystallizability of c-MYC and USP37, Xtalpred server [63] was used. Xtalpred predict the target protein crystallization potential on the basis of 12 parameters including length, PI, hydrophobicity, molecular weight and span of disordered regions.

#### 4.7.3. Protein Molecular Modeling

Homologous structures were selected on the basis of query coverage and sequence identity. Atomic coordinates of these structures were retrieved from the RCSB database using appropriate accession number and/or PDBid [61]. Molecular models of c-MYC, USP37 and disruptor peptide were developed using I-TASSER server [64], multiple models were constructed by I-TASSER and after structural and thermodynamic evaluations the best models for c-MYC and USP37 were selected. The structures were then further refined using MODELLER 9v8 156 [65] and Swiss PDB Viewer v4.0.2 [66]. Constructed models were refined and selected on the basis of their C-score (generated by I-Tasser), Ramachandran plot analysis using MolProbity [67], free energy estimation and structural alignment of Cα back bone of models with the template protein structures in terms of Root Mean Square Deviation (RMSD) in Å using Swiss-Pdb Viewer v4.0.2 [66].

#### 4.7.4. Molecular Docking

Molecular docking was conducted between c-MYC and USP37 and c-MYC and disruptor peptide using HADDOCK 2.2 [68]. The atomic coordinates of proteins were uploaded and run for molecular docking analysis employing default settings. HADDOCK assembled all docking simulations into clusters and ranked them according to the HADDOCK score on the basis of electrostatic energy, desolvation energy, Van der Waals energy, restraint violation energy and buried surface area. The cluster with least HADDOCK score was selected for further assessment. Binding affinity and different types of interactions like charged-charged, charged-polar, charged-apolar, polar-polar, polar-apolar and apolar- apolar were assessed using PRODIGY webserver [69]. All structures were visualized using DS visualizer 2016.

### 4.8. Statistical Analysis

For statistical analysis, GraphPad Prism v8.0 was used, the nature of data distribution was analyzed by Kolmogorov–Smirnov test. Mann–Whitney t-test was used to determine statistical significance of differences between the variables. The correlation between c-MYC and USPs expression in both subtypes of DLBCL were estimated by Spearman linear regression with 95% confidence interval and in all cases *p*-value ≤ 0.05 was considered statistically significant.

## 5. Conclusions

Our data reflect the role of and USP37 in maintaining the cellular turnover of the c-MYC in the ABC subtype of DLBCL. Furthermore, molecular structures of both proteins individually and in complex configuration have been predicted and disruptor peptide to block the complex formation has been proposed. The candidate disruptor peptide could be used for the therapeutic management of the ABC subtype of DLBCL after empirical validation.

## Data Availability

Not applicable.
